# High Resolution AMS Dates from Shubayqa 1, northeast Jordan Reveal Complex Origins of Late Epipalaeolithic Natufian in the Levant

**DOI:** 10.1038/s41598-017-17096-5

**Published:** 2017-12-05

**Authors:** Tobias Richter, Amaia Arranz-Otaegui, Lisa Yeomans, Elisabetta Boaretto

**Affiliations:** 1 Centre for the Study of Early Agricultural Societies, Department of Cross-Cultural and Regional Studies, University of Copenhagen, Karen Blixens Plds 8, Building 10, 2300 Copenhagen, Denmark; 2 Max-Planck-Weizmann Center for Integrative Archaeology and Anthropology, 76100 Rehovot, Israel and D-REAMS Radiocarbon Laboratory, 76100 Rehovot, Israel

## Abstract

The Late Epipalaeolithic Natufian (~14,600 − 11,500 cal BP) is a key period in the prehistory of southwest Asia. Often described as a complex hunting and gathering society with increased sedentism, intensive plant exploitation and associated with an increase in artistic and symbolic material culture, it is positioned between the earlier Upper- and Epi-Palaeolithic and the early Neolithic, when plant cultivation and subsequently animal domestication began. The Natufian has thus often been seen as a necessary pre-adaptation for the emergence of Neolithic economies in southwest Asia. Previous work has pointed to the Mediterranean woodland zone of the southern Levant as the ‘core zone’ of the Early Natufian. Here we present a new sequence of 27 AMS radiocarbon dates from the Natufian site Shubayqa 1 in northeast Jordan. The results suggest that the site was occupied intermittently between ~14,600 − 12,000 cal BP. The dates indicate the Natufian emerged just as early in eastern Jordan as it did in the Mediterranean woodland zone. This suggests that the origins and development of the Natufian were not tied to the ecological conditions of the Mediterranean woodlands, and that the evolution of this hunting and gathering society was more complex and heterogeneous than previously thought.

## Introduction

The lack of secure, continuous sequences of radiocarbon dates from Natufian sites has been a long running problem for researchers working on the Late Epipalaeolithic of the Levant^1–4^, particularly when it comes to the Early Natufian. Suitable sample material, especially charred botanical remains, is often lacking on Late Epipalaeolithic sites in the Levant, which has resulted in a patchy record. Most sites only have a small number of dates and many of these are problematic due to unclear sample provenience, use of unspecified or unidentified sample material, high standard deviations or because they were obtained a long time ago when radiocarbon dating was less well developed^1,5^. In addition, the uneven spread of research across the Levant has resulted in a skewed distribution of radiocarbon dates for the Natufian. The vast majority of dates were obtained from sites in modern-day Israel, while far fewer dates are available from the surrounding region. This situation continues to pose significant challenges to our understanding of the emergence and development of the Natufian in the Levant.

We report a new series of twenty-seven Accelerator Mass Spectrometry (AMS) dates from the Natufian site Shubayqa 1 in north-east Jordan. Unlike most other Natufian sites in the Levant Shubayqa 1 has exceptional preservation conditions for botanical remains that has enabled us to obtain the most detailed series of AMS dates yet available from any Natufian site in the southern Levant. Our results show that Shubayqa 1 was first occupied between ~14,400 − 14,200 cal BP (1σ) or ~14,600 − 14,200 cal BP (2σ). The Early Natufian occupation lasted for 300–600 years. The site was then abandoned for c. 900 years and occupied again in the late Natufian between ~13,300 − 13,100 cal BP (1σ) or ~13,300 − 13,000 cal BP (2σ). The site was then abandoned again and re-occupied ~12,160 − 11,650 cal BP (1σ) or ~12,380 − 11,500 cal BP (2σ). These dates are the first solidly dated Early-Late Natufian sequence outside the Mediterranean zone.

Between c. 14,600 − 13,600 cal BP Early Natufian groups in the Levant significantly modified their modes of subsistence and settlement patterns resulting in the appearance of large base-camp sites with stone-built dwellings, large numbers of ground stone artefacts, burials, art objects and small decorative personal ornaments such as shell, stone and bone pendants^6–12^. Early Natufian sites are concentrated in a ‘core zone’, which encompasses the Mount Carmel, Galilee, Jordan Valley and the adjacent Transjordanian highlands stretching south to the Petra and Hisma region^6,13–19^. The emergence of the Early Natufian is associated with the onset of warmer and moister climatic conditions of the Bølling-Allerød that led to an abundance in plant and animal resources enabling more permanent settlement and population growth^20,21^. The recovery of large numbers of ground stone tools, including pounding and grinding implements, glossy flint sickle-blades and storage pits, suggests that plants, and especially cereal grasses, were intensively exploited by these Late Epipalaeolithic communities^22–28^. These abundant cereal stands have been seen as a key resource that enabled groups to stay in the same location for longer periods of time. Thus, the Natufian has widely been described as one of the world’s earliest documented complex hunter-gatherer societies with a delayed return economy^7,29,30^. This switch from a mobile to a more sedentary way of life, and an implied reliance on wild plants, especially cereal grasses, have been described as crucial pre-adaptations that set these Late Epipalaeolithic communities on a path towards plant cultivation, village life and agriculture, which emerged during the early Holocene after ~11,700 cal BP^1,6–10,12,18,31–34^. Some scholars have, however, questioned the link between the Early Natufian and the Mediterranean zone. Olszewski^35–37^ argued that cereal stands would have been more ubiquitous in the steppe zone rather than the forest, while others have argued that the Natufian plant subsistence economy may have been more reliant on acorns than on cereals^38–40^. A recent review of the available archaeobotanical evidence for the Natufian also suggests that cereals were not an important component of the plant subsistence economy^41^.

The Early Natufian settlement and subsistence system is said to have undergone a significant crisis or re-adjustment with the onset of the Younger Dryas, which has been thought to have coincided with the Late Natufian. The cooler and drier conditions of the Younger Dryas placed stress on densely packed, semi- or fully sedentary groups in the Natufian ‘core’ zone, forcing population dispersal into more arid and peripheral ecotones, such as the steppe and desert zones of eastern Jordan and the Sinai and Negev^6–8,19,31,32,42–50^. In these areas, populations adopted a more mobile lifestyle based predominantly on hunting. In the Natufian core zone, however, populations retained a more sedentary settlement pattern. Recent work suggests, however, that the impact of the Younger Dryas on the environment of the Levant is now thought to have been less severe than previously thought^4,51,52^. Furthermore, the correlation between the Late Natufian and the onset of the Younger Dryas is now questionable^4,5,19,53–55^. Indeed, recent work suggests that the Early and Late Natufian phases had considerable overlap^55^.

## Shubayqa 1

**Figure 1:**
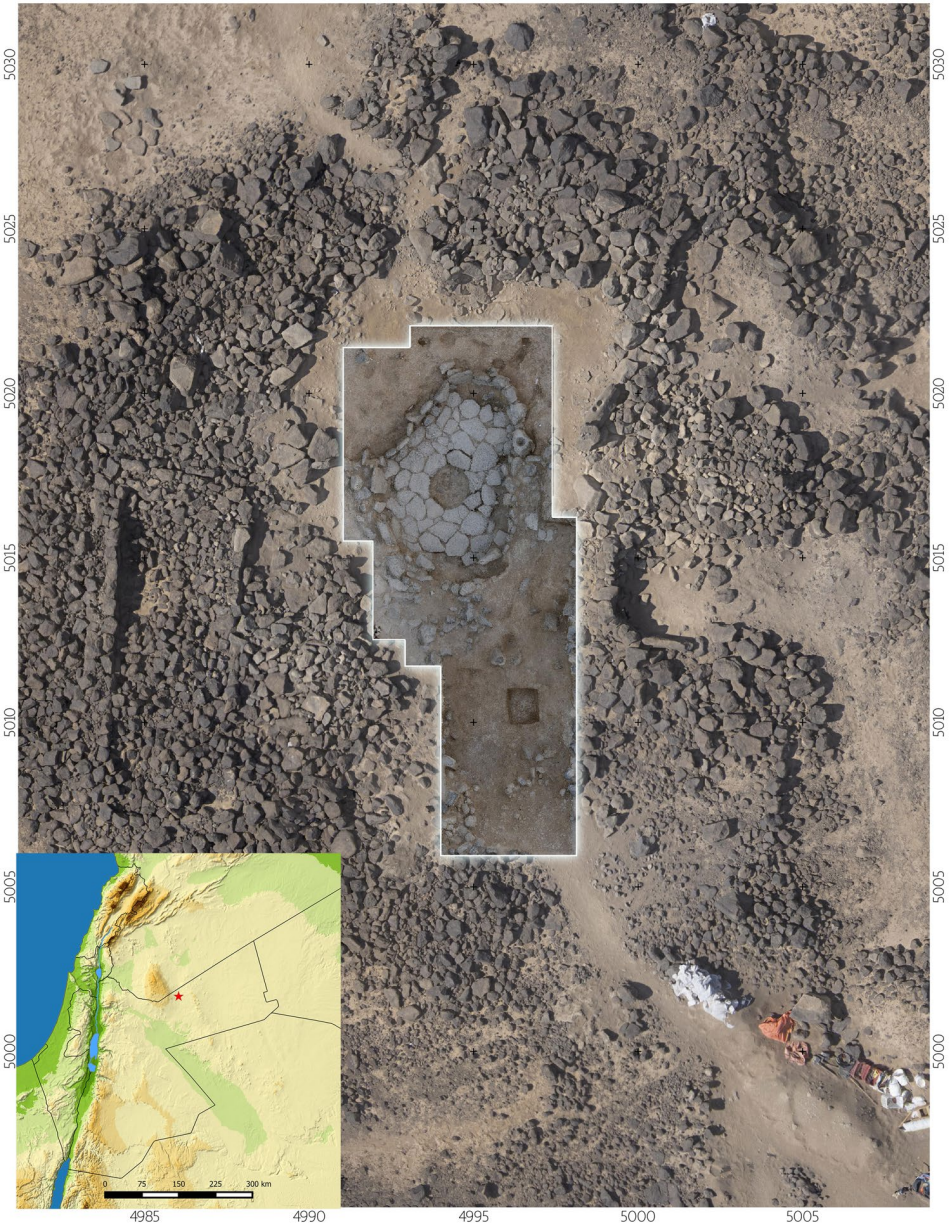
Aerial view of Shubayqa 1 showing the main excavation area A/B and location of Shubayqa 1 (insert bottom left). Map generated using data from Natural Earth (naturalearthdata.com) and processed using QGIS 2.18.13 (http://www.qgis.org/en/site/). Aerial photo by Alexis Pantos.

Shubayqa 1 (Lat 32.406437/Lon 37.228100) lies 132 km east-north-east of the Jordanian capital Amman, and 25 km north-north-east of the town of Safawi. It is situated in the al-Harrah volcanic field of the *Harrat al*-*Shamah*, also known as the Black Desert (Fig. 1). The settlement is immediately adjacent to a *Qa’* (Arabic for mudflat or playa) that extends over c. 12 km^2^ south of the site. Although this area receives less than 200 mm of mean annual rainfall today, allowing only for opportunistic dry-farming, the *Qa’* Shubayqa is inundated annually by surface run-off draining from Jebel Druze. Geomorphological and archaeological evidence in the form of charred remains of wetland plant species and numerous remains of waterfowl in the faunal assemblage from Shubayqa 1 suggest that the playa was likely a semi-permanent or permanent wetland during the late Pleistocene and possibly the early part of the Holocene^41,56,57^.

Betts carried out reconnaissance survey in the *Qa*’ Shubayqa area during the early to mid-1990s and reported the presence of a number of late Epipalaeolithic sites in the area, including Shubayqa 1 (54). A brief test excavation was carried out at the site in 1996, which revealed part of a structure. The site was relocated in 2009 and has been under excavation by the University of Copenhagen from 2012–2015^56–62^.

**Figure 2:**
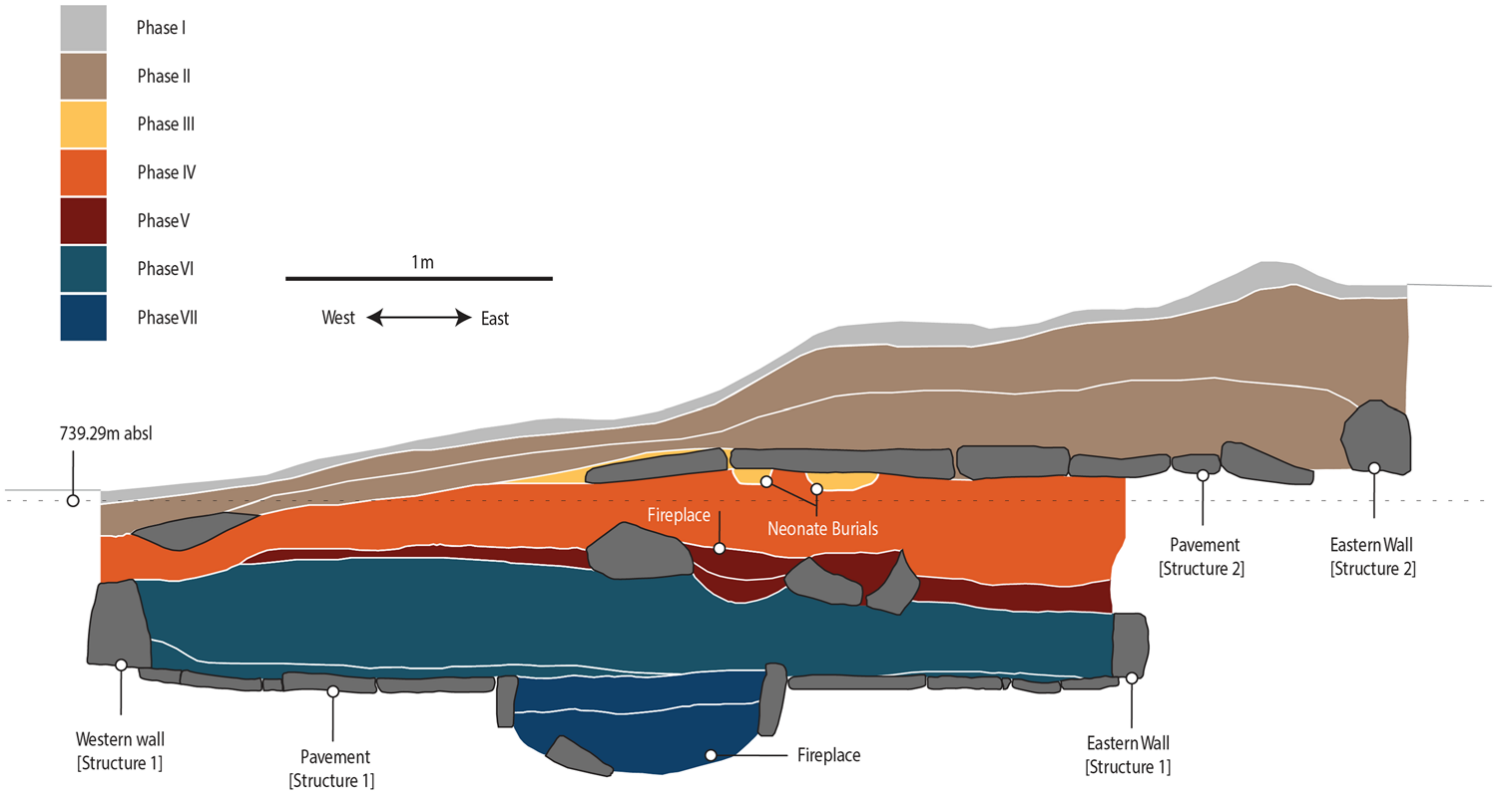
Schematic section of the stratigraphy of Shubayqa 1. Image prepared by Alexis Pantos.

Shubayqa 1 is centered on a low mound that rises 2–3 meters above the surrounding area (Fig. 1). The mound is composed of large basalt boulders, as well as natural and anthropogenic sediments. Its surface is littered with a dense scatter of chipped stone artefacts covering an area of approximately 2000 m^2^. Boulder mortars and other ground stone artefacts are also strewn across the surface of the mound. Excavations at the site have exposed an area of 94 m^2^. The majority (92 m^2^) was excavated in one main area (Area A/B, shown in Fig. 1), while the remainder was a small sondage excavated at the northern edge of the site (labeled Area C). In the main area, excavations exposed a 1.6 m thick stratigraphic sequence that included several buildings, middens, graves, installations, floors and other artefact bearing deposits. Based on stratigraphic observations seven major phases of occupation were distinguished (Fig. 2, for a detailed description of the excavation methods and stratigraphic phases see SI 1 and SI 2). The site has produced evidence for a number of stone buildings. Structure 1 and 2 are the best preserved examples. Structure 1 is a semi-subterranean oval shaped structure, with an exterior wall made up of a single row of upright standing stones, while the interior was covered by a flagstone paved floor, which contained a stone lined fire pit. The burial of an adult was recovered from the fill inside the construction cut of Structure 1’s exterior wall. Superimposed on top of Structure 1 was Structure 2. It is likewise a semi-subterranean structure, with a wall of upright standing stones and a stone pavement, which included several installations (a fireplace, cup-marked stones, ground stone mortar and grinding stones). Several burials of neonates and infants were interred beneath the stone floor of Structure 2. In 2015, the remains of two further, heavily deflated structures were exposed in an extension to Area A/B excavated to the south. Excavations have produced a wide range of material culture, including large numbers of flaked and ground stone artefacts, fauna, charred plant remains, worked bone, stone and marine shell beads, and incised objects.

## Results

**Figure 3:**
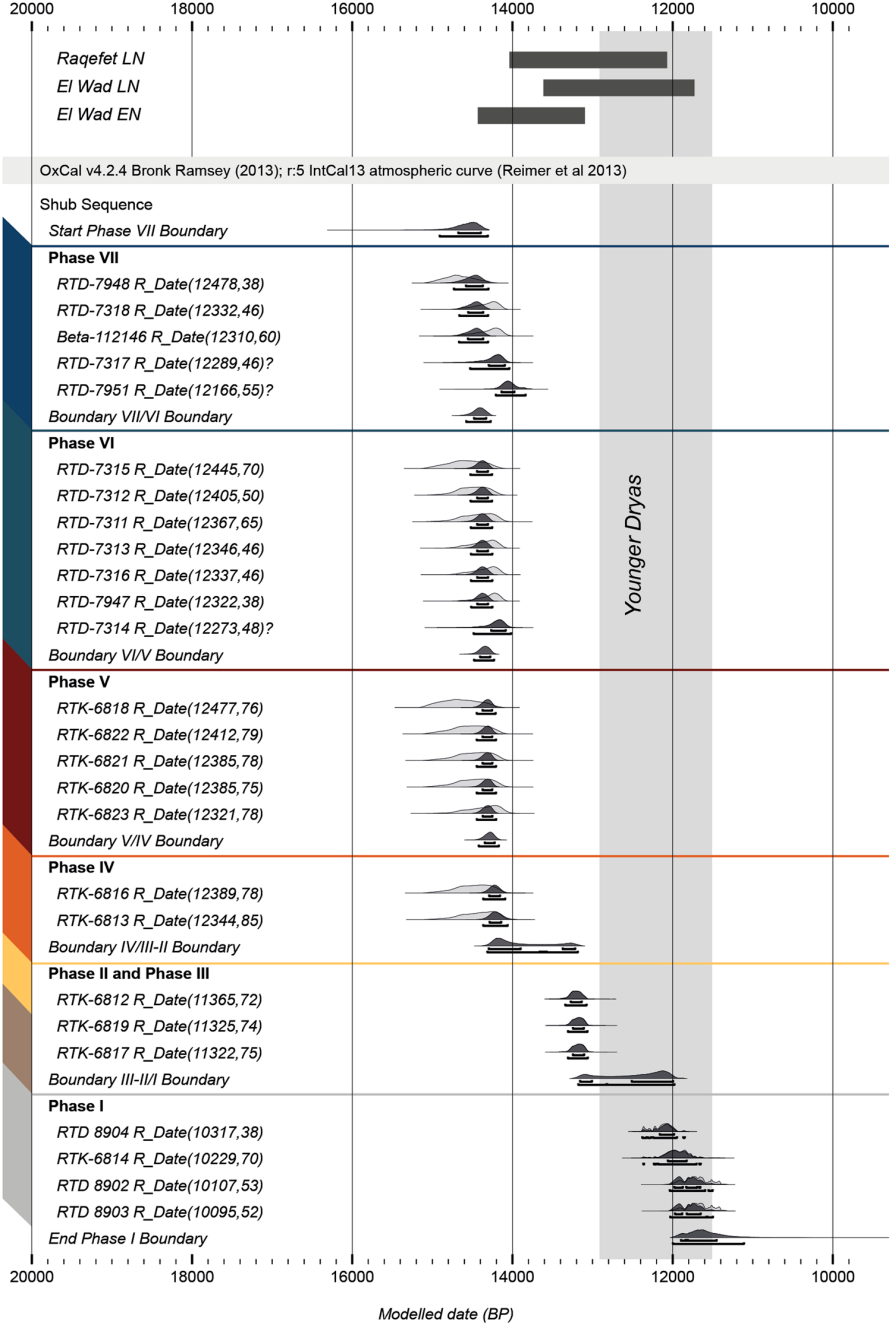
OxCal plot of the AMS dates from Shubayqa 1 sub-divided by stratigraphic phase. Dates for Late Natufian Raqefet, Late Natufian el-Wad and Early Natufian el-Wad shown at the top.

A total of twenty-seven samples of charred botanical material were dated at the D-REAMS Laboratory (see SI 2 for pre-treatment and laboratory procedures). One sample (RTK-7949, Phase 7) contained too little carbon for measurement and therefore did not return a date. Sample RTK-6815 from Phase 1, Area C, produced a date of 1,229 +/− 54 cal BCE, which suggests that the seed from which the date was obtained is intrusive. This date was omitted from the model and subsequent discussion. One date (Beta-112146) was obtained by Alison Betts after the 1996 excavations, but has not been published before. Owing to the meticulous recording of the original location of this sample, we were able to incorporate it into our sequence. We publish it here with the excavator’s permission. All charred plant material was identified to genus level and only short-lived plants or terminal branches of woody plants were used. Taking out the intrusive sample and the sample that did not produce a date, results in a sequence of twenty-six reliable dates with acceptable standard deviations from all phases of occupation at the site (Fig. 3, SI Table 1). We used the phase function in OxCal 4.3 to sub-divide the dates into seven phases based on our stratigraphic information and ran the model using the INTCal13 calibration curve (see SI 3 for the model). Although the dates could have been sub-divided further according to the site’s stratigraphic matrix, we felt that the broader overview afforded by the phase model made the presentation of the dates more straightforward. It is also the case that not all of the stratigraphic units within the same phase were necessarily superimposed, so that the phase model was the most straightforward approach to model the dates.

We obtained four dates from Phase 7 (RTK-7948, RTK-7318, RTK-7317 and RTK-7951). The model indicates poor agreement for three dates in this phase, which we therefore consider to be outliers. RTK-7951 and RTK-7317 produced dates that are too young for Phase 7, while RTK-7948 is too old. Why these dates are in poor agreement with the other dates from this phase is unclear. Although it is possible that the material is intrusive from a later phase, the contexts from which these two samples were obtained are very secure, one being the fill of a fireplace. There is little evidence for bioturbation at the site. Differences in ^14^C concentrations in these two samples, accidental contamination or other factors may explain why these samples produced slightly later dates. Beta-112146, originally obtained by Betts, fits well into the sequence, providing an independent confirmation of our results.

Six dates (RTK-7947, RTK-7316, RTK-7315, RTK-7314, RTK-7312 and RTK-7311) were obtained from Phase 6. After constraining them in the phase model these dates follow very quickly on those from Phase 7, suggesting that Structure 1 was abandoned only briefly and quickly re-used thereafter, possibly as a waste disposal area. This suggests that occupation shifted laterally, but the site was not abandoned.

Phase 5 is dated by six AMS determinations (RTK-7313, RTK-6823, RTK-6822, RTK-6821, RTK-6820 and RTK-6818). RTK-6818 has a low agreement value and appears to be too old for this phase. As for the dates in Phase 7 we are unclear about the reasons for this at present. The dates from this phase follow very quickly upon those of Phase 6, suggesting that occupation continued without interruption. In this phase, a fireplace was constructed more or less in the center of Structure 1, which appears to have been re-used by the occupants: the upper part of the exterior wall of Structure 1 would have been visible and stood out from the underlying sediment, whereas the flagstone pavement was already buried at this stage.

There are two dates from Phase 4: RTK-6813 and RTK-6816. Once more these follow quickly onto those of Phase 5 suggesting only brief use of the fireplace in Phase 5 and quick burial, probably associated with another lateral shift in occupation.

The model demonstrates that Phases 7–4 were laid down in relatively quick succession. These phases are bracketed between ~14,400 − 14,100 cal BP (1σ), representing a total span of occupation lasting ~300 years. At 2σ the dates are bracketed between ~14,600 − 14,000 cal BP. There is no apparent break between these phases, at least in terms of ^14^C dates. Although there appear to be brief episodes of site abandonment, as evidenced by the accumulation of an aeolian sediment at the end of Phase 7, the sequence of cultural deposition is otherwise continuous. Phases 7–4 fall within the time range of the Early Natufian.

The first real break in the radiocarbon sequence occurs between the end of Phase 4 and the start of Phase 3, which is marked by the construction and use of Structure 2. The hiatus between the end of Phase 4 and the start of Phase 3 is at minimum 820 years, at maximum 1100 years. The three dates from Phase 3 (RTK-6819) and 2 (RTK-6812 and RTK-6817) are very close in range, starting at around 13,390 cal BP and ending around 13,070 cal BP. While RTK-6819 was taken from the fill of a fire place inserted into the floor of Structure 2, RTK-6812 and RTK-6817 were taken from the midden infill of Structure 2. Their quick succession, once more suggests that Structure 2 was buried quickly and re-used as a waste disposal area, with occupation having shifted laterally to another part of the site. The dates correspond to the late Natufian phase, which is also confirmed by the dominance of non-Helwan backed lunates in the chipped stone assemblage (see below).

There is another break in occupation between Phase 2 and 1, which lasted between 1000–1100 years (1σ) or 700–1000 years (2σ). Secure contexts related to Phase 1 were only encountered in Area C (a 2 × 1 m sondage north of Area A/B), and the southern extension of Area A/B excavated in 2015. One date (RTK-6814) was obtained from Area C and three dates from the southern extension (RTD-8902, RTD-8903 and RTD-8904). Although these dates were obtained from two separate excavation areas, their close chronological proximity allows us to include them in the same phase. These four dates span the period from ~12,160 − 11,650 cal BP (1σ) or ~12,380 − 11,500 cal BP (2σ). The phase ends between ~11,900 − 11,450 cal BP (1σ) or ~11,990 − 11,080 (2σ). These dates fall firmly within the mid to late Younger Dryas.

To better correlate the Shubayqa 1 radiocarbon dates with the broader chronological framework of the Natufian we now consider one other key chronological marker for this cultural entity. Lunates (crescent shaped geometric microliths) have long been considered the most diagnostic aspect of the Natufian lithic industry and were originally used to define the Natufian as a distinct archaeological entity^63^. The morphology of the retouch along their backed edge has been shown to be chronologically sensitive: Helwan retouched lunates are characteristic of the Early Natufian; abruptly, bipolar or alternatingly backed lunates are characteristic of the Late Natufian^11,12,64–67^. In addition, it has been proposed that this change in mode of retouch was accompanied by a reduction in average lunate length^66,67^, although some of have argued that this is not applicable in some other parts of the Levant^68–70^. To correlate changes in lunate morphology with the radiocarbon dates obtained from Shubayqa 1 we examined a total of 429 complete and broken lunates from Phases 7–2 (SI Table 2). Lunates from Phase 1 have to date not been studied.

The 429 pieces are unevenly distributed across the stratigraphic sequence: 9% of the sample derive from Phase 7, 1.6% from Phase 6, 2.3% from Phase 5, 7.9% from Phase 4, and 79% derive from phase 2 and 3. Phase 2 and 3 were treated as one phase because there were very few lunates from Phase 3. The sample is therefore proportionally skewed towards Phase 2 and 3. Few lunates were available for study from Phase 6 and 5, leaving questions over the reliability of the data from these phases. However, it is important to point out that the numbers reported here are the total amount of lunates recovered from Phase 7−4.

**Figure 4:**
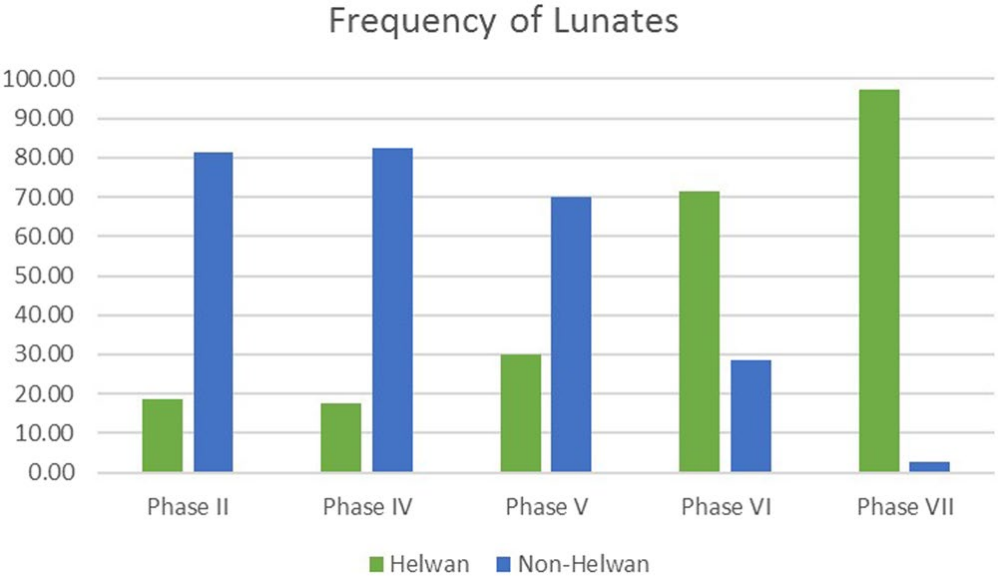
Frequency distribution of Helwan and non-Helwan backed lunates in the different stratigraphic phases at Shubayqa 1.

The data shows that Helwan lunates are typical in Phases 7 and 6 (Fig. 4). Their numbers decrease significantly in Phase 5, although overall lunate numbers in this phase are too low to be certain that these figures are reliable. However, in Phase 4, more lunates were found, and here non-Helwan lunates make up 82.3%. In Phase 2 & 3, non-Helwan lunates are also the majority.

Length and width measurements were only taken on complete specimens. The average length and width of Helwan backed lunates changes little from Phase 7 to Phase 2/3 (Fig. 4). Non-Helwan lunates are from Phases 6−2/3 are on average 2–3 mm shorter and 1–2 mm narrower, but no significant reduction in length or width within this group is evident. The overall mean length of Helwan lunates is between 19–22 mm, while the mean width is 7–8 mm. Non-Helwan lunates have a mean length of 17–19 mm, and an average width of 5–7 mm. This data compares well with lunate frequencies and length measurements from other Natufian sites in the southern Levant. The mean length of early Natufian lunates ranges between 18–28 mm, the width between 6–9 mm. In the late Natufian, lunates have a mean length of 15–20 mm and a mean width of 4–7 mm^53^.

The switch from Helwan to non-Helwan lunates in Phases 5 and 4 is of significance. Phase 5 has been dated to between ~14,300 − 14,100 cal BP, Phase 4 to between ~14,200 − 14,100 cal BP. This suggests that one of the key chronological markers for the Late Natufian may have appeared early at Shubayqa 1. Recent radiocarbon determinations for the Late Natufian phases at Raqefet Cave also suggested that the Late Natufian may have appeared earlier than previously thought and overlapped significantly with the Early Natufian^55^. However, it is important to point out that we recovered only few lunates from Phase 5. At the same time, some of the non-Helwan lunates in Phase 4 may be intrusive from Phase 3. During Phase 3 the pavement slabs of Structure 2 were sometimes lifted to inter human remains beneath the building. Although great care was taken during excavation to separate the fills of the graves from the surrounding sediment of Phase 4, some degree of admixture of material culture cannot be completely excluded. Nevertheless, the high frequency of non-Helwan lunates in the Phase 4 assemblage seems unlikely to be wholly the result of contamination from Phase 3. We therefore suggest that the observations are related to actual techno-typological changes.

## Discussion

Shubayqa 1 is the first well-dated, Early to Late Natufian site with significant architectural remains identified outside the Mediterranean woodland zone in the southern Levant. Measuring more than 1,000 m^2^ in extent, with impressive architecture, thick occupation deposits, burials, a large assemblage of ground stone artefacts, bone tool production and numerous bone and shell pendants, it fulfills all the criteria of a Natufian ‘base-camp’. The chronology of Shubayqa 1 poses a number of questions for our understanding of the emergence and development of the Late Epipalaeolithic Natufian.

The sequence of AMS dates presented here, suggests that Shubayqa 1 was established as early as ~14,400 cal BP (1σ) or possibly ~14,600 cal BP (2σ). Cumulatively, the Shubayqa 1 dates are earlier than the sequence of Early Natufian dates recently published for el-Wad Terrace^3^. Although there are three older dates from el-Wad Terrace, the oldest is dismissed by Weinstein *et al*.^3^ as out of sync with the other dates in the sequence. The two other dates from what Weinstein-Evron *et al*. termed the ‘early Early Natufian’^71,72^ are the only two dates from a Natufian site that predate 15,000 cal BP. These two dates have underlined the primacy of the Mount Carmel region as the core region of the Early Natufian. We note, however, that these two dates have not been confirmed by the recent dates obtained from el-Wad or dates from other sites in the same area. Furthermore, the charcoal used as sample material for both dates was not identified to genus level and has not been described in detail in publication. Recent work has highlighted the crucial importance of using short-lived plant species or short-lived plant elements for ^14^C dating^73–76^. Given that the early dates from el-Wad cave chamber III appear to be somewhat earlier than all other available dates, and given the unclear characteristics of the charcoal that was dated, the reliability of these dates is not given. Thus, a more prudent approach would seem to suggest that the Early Natufian begins at ~14,600 cal BP. This means that Shubayqa 1 was first established soon after or as early as the Early Natufian sites in the Mediterranean ‘core zone’. This suggests either a rapid spread of the Early Natufian across the region or a more multi-regional emergence of Early Natufian traits. In the Azraq Basin, previous fieldwork has established a long continuity of Epipalaeolithic settlement^77–85^. It is now evident that this continuity extends to the Late Epipalaeolithic Natufian as well, and that significant developments occurred in the Azraq Basin. This ties in with evidence for extensive regional interaction between pre-Natufian Epipalaeolithic communities in the southern Levant, as evidenced by similarities in chipped stone toolkits as well as the exchange of marine shell pendants^81,86^. This pattern of exchange also continued in the Late Epipalaeolithic, as evidenced by the numerous dentalium and other shell pendants found at Shubayqa 1.

The evidence presented here also shows that the Natufian was not tied or confined to the Mediterranean woodland zone. Given the abundant rainfall the Jebel Druze still receives today, it has been argued that this area was likely populated by oak-terebinth-Rosaceae park-woodland surrounded by terebinth-almond woodland-steppe with extensive stands of wild wheats and ryes during the Bølling-Allerød^87^. The botanical evidence from Shubayqa 1 suggests that Early Natufian hunter-gatherers in eastern Jordan did not rely on cereals or nuts as their primary source of plant food, but club-rush tubers^41^. This indicates that the early Natufian settlement and subsistence system – characterized by increased sedentism and specialized exploitation of particular plant resources – was more variable than previously known and that people took advantage of a much wider range of ecotones.

The ^14^C sequence from Shubayqa 1 also reveals that the switch from Helwan-backed lunates to non-Helwan lunates occurred in Phase 4 or 5. By combining the various dates of this phase with the previous and subsequent phase, it appears likely that this switch occurred between ~14,300 and 14,100 cal BP, corroborating evidence for the early appearance of the Late Natufian lithic industry at ~14,000 cal BP from Raqefet^55^. It appears that the Late Natufian may have emerged just as early in the *Harra* as in the Mediterranean Levant and co-existed along early Natufian lithic industries between ~14,300 to as late as ~13,600 cal BP. This also means that the dating of other Natufian sites in eastern Jordan (and perhaps elsewhere) has to be reconsidered. Many of these sites were assigned to the period after 13,200 cal BP solely on the basis of lithic typology^19,88^. In the light of the evidence from Shubayqa 1 many of these sites (e.g. Khallat Anaza, Jebe Subhi) could date as early as ~14,300 cal BP. Azraq 18, which has a mixed assemblage of Helwan and non-Helwan type lunates, is probably comparable to Phase 6 and 5 at Shubayqa 1.

The dates for Phase 1 at Shubayqa 1 also indicate a substantial presence of late Natufian groups in eastern Jordan during the mid to late Younger Dryas. Although there is a long gap in the sequence between Phase 2 and Phase 1, covering the earlier part of the Younger Dryas, our evidence suggests that the area was not completely abandoned for all of the Younger Dryas. More importantly, although there is a gap, it can be argued that there is nevertheless a certain degree in continuity, as people returned to the same settlement location even after a long interruption.

In the light of this new evidence, the concept of the Natufian ‘core’ requires critical evaluation. Evidence presented here and from other regions in the Levant^15,89–93^ indicates that substantial Early Natufian base-camp type sites existed outside the Mediterranean zone as early as 14,600 − 14,400 cal BP and that the Natufian did not arrive in eastern Jordan as part of a dispersal from the west during the Younger Dryas. This apparently more heterogenous process suggests that there was not one pathway from complex hunting and gathering to food production during the transition from the Late Epipapaleolithic to the early Neolithic in the Levant. If people pursued multiple plant subsistence strategies, in variable ecotones and regions, more or less at the same time, it suggests that there were also multiple routes towards the emergence of the Neolithic economy. This underscores, in our view, that the emergence of plant cultivation, animal domestication and the ‘Neolithic way of life’ was a highly variable and more complex process that cannot be explained on the basis of single-cause models^94–97^.

## Electronic supplementary material

Supplementary Information

Radiocarbon Dates

Lunate Analysis
