# Supplementary material for: High Resolution AMS Dates from Shubayqa 1, northeast Jordan Reveal Complex Origins of Late Epipalaeolithic Natufian in the Levant
Source: Sci Rep. 2017 Dec 5;7:17025. doi: 10.1038/s41598-017-17096-5 (PMC5717003; doi:10.1038/s41598-017-17096-5)
Supplement: Supplementary file 1 — Supplementary Information [file 41598_2017_17096_MOESM1_ESM.pdf]

High Resolution AMS Dates from Shubayqa 1, northeast Jordan Reveal Complex Origins of Late Epipalaeolithic Natufian in the Levant

Tobias Richter[1]*, Amaia Arranz-Otaegui[1], Lisa Yeomans[1] and Elisabetta Boaretto[2]*

[1] Centre for the Study of Early Agricultural Societies, Department of Cross-Cultural and Regional Studies, University of Copenhagen, Karen Blixens Plds 8, Building 10, 2300 Copenhagen, Denmark

[2] Max-Planck-Weizmann Center for Integrative Archaeology and Anthropology, 76100 Rehovot, Israel and D-REAMS Radiocarbon Laboratory, 76100 Rehovot, Israel

*Corresponding authors: richter@hum.ku.dk, Elisabetta.Boaretto@weizmann.ac.il

- SUPPLEMENTARY INFORMATION -

Methods

Excavations at Shubayqa 1 were carried out using a 1 x 1 meter grid system to ensure a high accuracy in recording the spatial distribution of finds. Sediments were excavated using a combined system of singe-context recording and excavation in arbitrary levels in each square meter. Thus, thick deposits were sub-divided into smaller excavation spits, but natural boundaries of sediments were always respected. Spoil from the excavations was dry-sieved on site using a 1 x 1 mm mesh. Sediment samples were collected from all excavated contexts for flotation, phytolith and pollen analysis. Wherever possible samples for charred plant remains for ^{14}C dating were collected in the field and placed into sterile sample containers. Alternatively, they were taken from bulk botanical samples in those cases were samples could not be collected on-site from some contexts or where those samples proved unsuitable for dating. The provenience of every sample collected during actual excavation was recorded noting their position on the site grid and elevation above sea level. Where possible charred plant remains were identified to genus or species level. Even where this was not possible only short-lived parts of plants (seeds or twigs) were used for dating to avoid use of old wood. Dating was carried out at the D-REAMS (Dangoor REsearch Accelerator Mass Spectrometer) Radiocarbon laboratory.

Samples were pre-treated using the acid-base-acid method, aimed to remove all the contaminants from the sample material, following the procedure in Boaretto et al 2009. Few animal bones were tested for collagen presence using the Fourier Transform Infrared Spectroscopy to analysis the acid insoluble fraction and test the presence of collagen for possible radiocarbon dating following the procedure in Yizhaq et al 2005. Unfortunately no bones have indicated that collagen was preserved. Dates were calibrated in OxCal 4.3 using the IntCal13 calibration curve. We provide dates at the 1σ range of 68.2% probability throughout the text. For the 2σ range please see the supplementary files. Using the position of each date in the stratigraphic matrix of the site, we were able to further constrain the dates using the phase function in OxCal 4.3 to create a Bayesian model for Shubayqa 1. To examine changes in lithic typology we recorded all complete and broken lunates from all the phases represented in the main stratigraphic sequence at the site. All lunates were categorized as either with or without Helwan retouch. Length and width measurements were taken using calipers on all complete lunates.

References Cited

E. Boaretto, X. Wu, J. Yuan, O.Bar-Yosef, V. Chu, Y. Pan, K. Liu, D. Cohen, T. Jiao, S. Li, H. Gu, P. Goldberg and S. Weiner. 2009. Radiocarbon Dating of Charcoal and Bone Collagen associated with the Early Pottery at Yuchanyan Cave, Hunan Province, China. *Proceedings of National Academy of Science* 106 (24), 9595-9600

M. Yizhaq, G. Mintz, I. Cohen, H. Khalally, S. Weiner and E. Boaretto. 2005. Quality Controlled Radiocarbon Dating of Bones and Charcoal from the early Pre-Pottery Neolithic B (PPNB) of Motza (Israel). *Radiocarbon* 47(2), 193-206

Shubayqa 1 Description and Overview of Stratigraphic Phases

Phase	Description	Radiocarbon Dates
7	earliest phase; associated with the construction and use of Structure 1. Finds related to the occupation and use of Structure 1 were recovered from the building's stone-lined fireplace, as well as the construction cut of the structure's exterior wall. The two fills of the fireplace were particularly dense in material culture, faunal remains and especially charred plant remains.	RTD-7951<br>Beta-112146<br>RTD-7317<br>RTD-7318<br>RTD-7948
6	begins after the abandonment of Structure 1. A thin layer of aeolian sediment accumulated above the floor of Structure 1 along the western wall. A thick clayey silt deposit accumulated above this aeolian layer, which contained few finds. Although finds from this deposit were rare, it appears to have an organic and anthropogenic character. This suggests that occupation at the site may have shifted laterally to another part of the site, but that the site continued to be used	RTD-7947<br>RTD-7313<br>RTD-7311<br>RTD-7312<br>RTD-7314<br>RTD-7316<br>RTD-7315
5	intermediate phase of occupation reusing the outer wall of Structure 1; construction of a fireplace, stone pavement and trampled surface. The fireplace contained two fills both of which were rich in charred plant remains, fauna and chipped stone. Burials were placed beneath the stone pavement.	RTK-6818<br>RTK-6820<br>RTK-6821<br>RTK-6822<br>RTK-6823
4	gradual burial of the Phase 5 fireplace and trampled surface by the accumulation of a c. 0.4 m thick silty clay deposit above the fireplace, which has a low to average finds density. Similar to Phase 6, it is suggested that occupation at the site continued, but shifted to another part of the site.	RTK-6813<br>RTK-6816
3	begins with the construction of Structure 2 and includes its use. It ends with the abandonment of the building. The boundary between Phase 2 and 3 – abandonment of structure and start of midden formation – is indistinct and difficult to separate. During this phase the pavement slabs of Structure 2 were repeatedly lifted and the remains of eleven neonates and infants were interred beneath and on top of the floor.	RTK-6819
2	consists of a midden deposit which yielded large assemblages of material culture and fauna. The midden overlay the flagstone pavement of Structure 2. Limited post-Epipalaeolithic material culture occurs in the upper-most part of the deposit, suggesting some recent disturbances; however, they are absent from the lowest layers of the midden. In addition to chipped stone and faunal remains, many ground stone tools were found lying on top of the pavement of Structure 2, although it is unclear whether they were left in situ after Structure 2 fell out of use or whether they accumulated inside the structure as part of waste disposal and general midden accumulation. Disarticulated human remains were also found lying on top of the pavement within the midden.	RTK-6812<br>RTK-6817
1	corresponds to the occupation exposed in Area C. In this small 1x2m sounding a charcoal rich deposit was located that produced a limited number of artefacts and other finds, and may represent an ephemeral occupation of the site. However, exposure to date has been too limited to fully understand the relationship of this deposit to other parts of the site.	RTK-6814<br>RTK-6815

OxCal Model Code

```
Options()
 {
  BCAD=FALSE;
 };
 Plot()
 {
  Sequence(Shub 1)
  {
   Boundary("Start Phase 7");
   Phase ("7")
   {
    R_Date("RTD-7948", 12478, 38)
    {
    };
    R_Date("RTD-7318", 12332, 46)
    {
    };
    R_Date("Beta-112146", 12310, 60)
    {
    };
    R_Date("RTD-7317", 12289, 46)
    {
     Outlier();
    };
    R_Date("RTD-7951", 12166, 55)
    {
     Outlier();
    };
   };
   Boundary("Boundary 7/6");
   Phase("6")
   {
    R_Date("RTD-7315", 12445, 70)
```

```
 {
 };
 R_Date("RTD-7312", 12405, 50)
 {
 };
 R_Date("RTD-7311", 12367, 65)
 {
 };
 R_Date("RTD-7313", 12346, 46)
 {
 };
 R_Date("RTD-7316", 12337, 46)
 {
 };
 R_Date("RTD-7947", 12322, 38)
 {
 };
 R_Date("RTD-7314", 12273, 48)
 {
  Outlier();
 };
};
Boundary("Boundary 6/5");
Phase ("5")
{
 R_Date("RTK-6818", 12477, 76)
 {
 };
 R_Date("RTK-6822", 12412, 79)
 {
 };
 R_Date("RTK-6821", 12385, 78)
 {
 };
 R_Date("RTK-6820", 12385, 75)
```

```
 {
 };
 R_Date("RTK-6823", 12321, 78)
 {
 };
};
Boundary("Boundary 5/4");
Phase ("4")
{
 R_Date("RTK-6816", 12389, 78)
 {
 };
 R_Date("RTK-6813", 12344, 85)
 {
 };
};
Boundary("Boundary 4/3-2");
Phase("2 and 3")
{
 R_Date("RTK-6812", 11365, 72)
 {
 };
 R_Date("RTK-6819", 11325, 74)
 {
 };
 R_Date("RTK-6817", 11322, 75)
 {
 };
};
Boundary("Boundary 3-2/1");
Phase ("1")
{
 R_Date("RTD 8904", 10317, 38)
 {
 };
```

```
    R_Date("RTK-6814", 10229, 70)
    {
    };
    R_Date("RTD 8902", 10107, 53)
    {
    };
    R_Date("RTD 8903", 10095, 52)
    {
    };
   };
  Boundary("End Phase 1");
 };
};
```
